# Gluten-Free Crackers Based on Chickpea and Pumpkin Seed Press Cake Flour: Nutritional, Functional and Sensory Properties

**DOI:** 10.17113/ftb.60.04.22.7655

**Published:** 2022-12

**Authors:** Jelena Tomić, Dubravka Škrobot, Ljiljana Popović, Tamara Dapčević-Hadnađev, Jelena Čakarević, Nikola Maravić, Miroslav Hadnađev

**Affiliations:** 1 University of Novi Sad, Institute of Food Technology, Bulevar cara Lazara 1, 21000 Novi Sad, Serbia; 2 University of Novi Sad, Faculty of Technology, Bulevar cara Lazara 1, 21000 Novi Sad, Serbia

**Keywords:** gluten-free crackers, chickpea flour, pumpkin seed press cake flour, glycaemic index, total phenolic content, protein content

## Abstract

**Research background:**

Despite the growing trend of the gluten-free market and the presence of a wide range of gluten-free products, there are still some shortcomings in nutritional and sensory quality of these products. The commercially available gluten-free products are characterised as products of inferior nutritional quality, particularly in terms of protein and dietary fibre content and with high glycaemic index. On the other hand, from a sensory point of view, gluten-free products usually have inappropriate textural and mechanical properties, poor mouthfeel and flavour. This is a consequence of the limiting choice of raw materials that mainly possess large amount of carbohydrate components.

**Experimental approach:**

Chickpea flour and two types of pumpkin seed press cake flour (virgin and cold pressed), at two substitution mass fractions (20 and 35%), were blended to produce gluten-free crackers without the presence of conventional gluten-free starch-rich ingredients. This study aims to investigate the effect of these non-conventional flours on nutritional and physicochemical properties, sensory acceptability, antioxidant activity and glycaemic index of crackers.

**Results and conclusions:**

All produced crackers can bear nutritional claims ’high fibre’, ’source of protein’ and ’source of minerals’. Replacing chickpea flour with pumpkin seed press cake flour increased protein and total phenolic content and enhanced antioxidant activity. The selected combination of raw materials allows the production of gluten-free crackers with a moderate glycaemic index. Besides nutrient content, the addition of cold-pressed flour increased overall sensory acceptability, noticeably improving taste and flavour scores compared to the control and crackers with virgin pumpkin seed flour.

**Novelty and scientific contribution:**

To the best of our knowledge, there is no study investigating the use of chickpea and pumpkin seed press cake flour blend without using conventional gluten-free flour and starch. The used non-conventional flour represents complementary raw materials in terms of protein quality and valuable alternatives to produce nutrient-rich, health-promoting gluten-free crackers with reduced glycaemic response and acceptable sensory properties.

## INTRODUCTION

In the circumstances of constant population growth and modern diet trends, the food industry faces great challenges in its desire to balance market dynamics and sustainable supply of various nutritious foods. The baked products (bread, biscuits and crackers) as the core of the consumer segment in the food industry are particularly susceptible to modifications and constant development in order to improve their nutritional composition without compromising consumer acceptability. In this regard, the utilization of various food ingredients that will provide adequate nutritional effects becomes the priority for both the food scientist and the food industry.

For the new food ingredients, especially in the case of gluten-free products, it is desirable to provide dual benefit of enhancing nutritional and retaining or improving technological quality of final products. Since gluten-free products are recognized as products with inferior nutritional quality, many studies with different technological and compositional approaches have been dedicated to the fortification of this category of food (*1*–*4*).

The lower dietary fibre and complex carbohydrate contents along with higher glycaemic index (GI) are considered as main drawbacks of gluten-free diet (*5*). In addition, a lower protein content than their gluten-containing counterparts has also been reported (*6*, *7*).

A compromise between the health benefits of a gluten-free diet and its nutritionally balanced profile and technological quality is very difficult to achieve. The reason for this is certainly the limiting choice of raw materials that provide the functional properties characteristic of gluten. By reviewing the literature data, but also by looking at commercially available gluten-free products, it is evident that rice and corn flour, as well as starch of different origins, are the most common ingredients of gluten-free products (*5*, *8*). Their selection is based primarily on the fact that they are of neutral taste and acceptable colour, good digestibility and have hypoallergenic properties (*9*, *10*). On the other hand, as a consequence of the large share of carbohydrate components in these raw materials, gluten-free products are usually characterized by poorer sensory quality, especially in terms of textural and mechanical properties (crumbling texture, poor mouthfeel and flavour) (*11*). Taking into consideration all the mentioned shortcomings of gluten-free products, reformulation of food by introducing a wide range of unconventional gluten-free flours has become a common practice. Evidence about considerable amounts of nutrient-rich ingredients found in chickpea has focused a lot of attention on redesigning conventional foods containing this raw material, mainly in the form of protein flour (*1*, *12*). Chickpea is a valuable source of proteins with a good amino acid profile (high lysine content), complex carbohydrates (dietary fibre, resistant starch and oligosaccharides), important vitamins and minerals (B vitamins, folates and iron), as well as antioxidants and polyphenols (*13*). Additionally, the benefit of the usage of chickpea flour as an alternative raw material in gluten-free products is reflected through the slow release of glucose from starch, which contributes to the lower glycaemic index (*2*). In the view of the environmental sustainability and minimisation of food waste, agro industrial by-products present a real impetus for the development of additional health-promoting ingredients. Pumpkin seed press cake, a by-product of oil production, is a food resource that offers various health benefits. In addition to fibre, pumpkin seed press cake is a source of proteins, essential fatty acids, polyunsaturated fatty acids, omega 3, 6 and 9 fatty acids, as well as significant amounts of vitamins and minerals (*14*–*16*). Regarding the protein quality, this raw material has a suitable amino acid profile. Compared to soya bean meal, it contains higher amounts of most essential amino acids (except lysine) (*17*).

From the nutritional point of view, chickpea and pumpkin seed press cake flours are complementary raw materials in terms of protein quality. Most studies that have investigated these raw materials focused on their valorisation and potential as gluten-free substitutes of conventional gluten-free flours in the production of bakery products. However, there are no published data on the use of chickpea and pumpkin seed press cake flour blend in gluten-free bakery products without the addition of any conventional gluten-free flour and starch. Therefore, the objective of this study is to investigate the possibility of using the aforementioned raw materials as base ingredients for the production of sensorially acceptable and nutritionally improved gluten-free crackers with respect to the content of protein and dietary fibre. Additionally, fatty acid content, glycaemic index, polyphenolic content and antioxidant activity were determined and discussed. Moreover, the impact of pumpkin seed press cake flour treatment history on the quality of the obtained crackers was evaluated.

## MATERIALS AND METHODS

### Raw materials

Chickpea flour was locally produced and obtained from agricultural household Mirilov, Bačko Gradište, Serbia. Its moisture, protein, fat, ash and carbohydrate (calculated by difference) mass fractions were 9.27, 21.52, 4.78, 3.29 and 61.14 g/100 g, respectively.

Samples of hulled pumpkin (*Cucurbita pepo* L) seed press cake, a by-product of the pumpkin cold-pressed and virgin pumpkin oil, were obtained from Suncokret d.o.o, Hajdukovo, Subotica, Serbia, which uses traditional mechanical oil pressing technology. The pumpkin seeds used for the production of virgin oil went through thermal processing prior to oil extraction. The obtained press cake samples were ground to obtain flour. The roasted pumpkin seed press cake flour had moisture, protein, fat, ash and carbohydrate (calculated by difference) mass fractions of 2.70, 56.16, 13.92, 8.59 and 18.63 g/100 g, respectively, while the cold-pressed pumpkin seed cake flour had 8.27, 56.49, 16.79, 7.57 and 10.88 g/100 g, respectively. Vegetable fat (palm oil-based, melting temperature 34-37 °C) was obtained from oil factory Dijamant (Zrenjanin, Serbia). Salt, sodium bicarbonate, ammonium bicarbonate and powdered sugar were purchased in a local food store.

### Preparation of gluten-free crackers

Five different formulations of gluten-free crackers were produced under the same processing conditions by mixing chickpea flour with virgin or cold-pressed pumpkin seed press cake flour in different ratios (Table 1). The other ingredients were used in the same proportions. Chickpea flour, pumpkin seed press cake flour, salt and baking powder were mixed together. The dough was prepared using the following procedure: vegetable fat was mixed with soy lecithin for 2 min, and then water was added and thoroughly blended to obtain homogenous mixture. Finally, all powdery ingredients were added together and mixed for additional 3 min. The dough was laminated immediately after preparation on pilot scale dough laminator (Macpan, Thiene, Italy) to the desired thickness (*b*=3.0 mm). Cracker samples were shaped using a circular cutter (*d*=45 mm) and baked at 190 °C for 11 min in a laboratory oven (MIWE gusto® CS, Arnstein, Germany). The crackers were produced in four batches where each batch yielded 20 crackers. The obtained gluten-free crackers were left to cool down at room temperature for 1 h and then they were hermetically stored for analysis.

**Table 1:** Formulation of crackers

Sample/raw material	*w*/%							
Chickpea flour	Virgin pumpkin seed press cake flour	Cold-pressed pumpkin seed press cake flour	Vegetable fat	Salt	Soy lecithin	Baking powder	Water
Control	100	-	-	25	2.5	0.5	0.5	40
20VFC	80	20	-	25	2.5	0.5	0.5	40
35VFC	65	35	-	25	2.5	0.5	0.5	40
20CFC	80	-	20	25	2.5	0.5	0.5	40
35CFC	65	-	35	25	2.5	0.5	0.5	40

Control=chickpea crackers, 20VFC and 35VFC=crackers with 20 or 35% of chickpea flour replaced with pumpkin seed press cake flour obtained after virgin pumpkin oil extraction, 20CFC and 35CFC=crackers with 20 or 35% chickpea flour replaced with pumpkin seed press cake flour obtained after cold-pressed oil extraction

### Proximate composition

Proximate composition of raw materials and crackers including protein (AOAC method 920.87), fat (AOAC method 922.06), ash (AOAC method No. 923.03) and moisture mass fractions (AOAC method No. 925.09) was determined by AOAC standard methods of analysis (*18*). Total dietary fibre content of the obtained crackers was determined using the Megazyme Total Dietary Fiber Assay Kit (Neogen, Lansing, MI, USA) by a method adopted from AOAC method 985.29 (*18*) and AACC method 32–07 (*19*). Available carbohydrate content was obtained by difference, by subtracting the sum of mass of water, protein, fat, ash and dietary fibre in g per 100 g of sample. The total energy was calculated using the European Regulation No. 1169/2011 (*20*).

### Mineral content

Mineral content (Zn, Fe, Mg, K, Na, Ca) was determined by atomic absorption spectrophotometry (AOAC Method 984.27) on a Varian Spectra AA 10 (Varian Techtron Pty Ltd., Mulgvare Victoria, Australia) (*18*).

### Fatty acid composition

The procedure published by Pojić *et al*. (*21*) was used to determine fatty acid profile. Total lipids were extracted with a solution *φ*(chloroform, methanol)=2:1 and the obtained extracts were dried by vacuum evaporation (Rotavapor R-210/215; Büchi AG, Flawil, Switzerland) at 40 °C. The residue obtained after the evaporation of solvent under steam of nitrogen was weighed. The extracted lipids were transformed in fatty acid methyl esters using 14% trifluoroborane in methanol. The samples were analysed by Agilent 7890A gas chromatography with a flame-ionization detector, auto injection module for liquid samples and fused silica capillary column DB WAX (30 m×0.25 mm, 0.50 µm; Agilent Technologies, Santa Clara, CA, USA). As a carrier gas, helium was used with purity over 99.9997% and flow rate of 1.26 mL/min. Fatty acids were identified by comparing the retention times with standards from Supelco 37 Component FAME Mix (Sigma-Aldrich, Merck, St. Louis, MO, USA). All analyses were performed in duplicates and the results were expressed as a percentage of each fatty acid in total fatty acids.

### Total phenolic content

Total phenolics from the samples were extracted by the method of Świeca *et al.* (*22*). Briefly, crackers were ground (1.0 g) and mixed with 20 mL of phosphate-buffered saline (PBS, pH=7.4) for 1 h at room temperature. The procedure was repeated twice. The extracts were centrifuged at 9200×*g* (Sorvall® RC-5B refrigerated superspeed centrifuge; Du Pont Instruments, Wilmington, DE, USA) for 15 min and the resulting supernatants were pooled and stored at 4 °C.

Total phenolic content of PBS extracts was determined spectrophotometrically using Folin‐Ciocalteu reagent method described by Singleton *et al.* (*23*). The extract (0.1 mL) was diluted with distilled water (7.9 mL). Folin‐Ciocalteu reagent (0.5 mL) and 20% sodium carbonate solution (1.5 mL) were added to the reaction mixture. The mixture was allowed to stand for 60 min and the absorbance was measured at 750 nm (T80 UV–Vis spectrophotometer; PG Instruments, Lutterworth, UK). Gallic acid was used as a standard and the results were expressed in mg of gallic acid equivalents (GAE) per g of sample.

### Antioxidant activity

The radical scavenging activity was determined by the ABTS scavenging activity assay described by Popović *et al.* (*24*), with some modifications. Briefly, an aliquot of 30 μL of the PBS extract was mixed with 3 mL of a daily prepared ABTS solution (*A*=0.7±0.02). The absorbance was measured at 734 nm (T80 UV–Vis spectrophotometer; PG Instruments) after 10 min. The activity was expressed in mM Trolox equivalents (TE) per g of sample.

### In vitro starch digestion rate and predicted glycaemic index

Predicted glycaemic index of crackers was determined by method of Molinari *et al.* (*25*), with some modifications. The ground crackers (200 mg) were mixed with 20 mL HCl-KCl buffer (0.01 M, pH=1.5) and 0.2 mL of pepsin solution (1 g in 10 mL HCl-KCl buffer). The mixture was incubated for 60 min in a shaking water bath at 40 °C. After pepsin digestion, the total sample volume was adjusted to 60 mL with phosphate buffer (0.2 M, pH=6.9). Then, 10 mg of α-amylase were added and incubated for 3 h in a shaking water bath at 37 °C. Aliquots (1 mL) at 0, 10, 20, 30, 60, 90,120, 150 and 180 min were obtained from each sample and incubated at 100 °C for 5 min to inactivate the enzyme. After incubation, 250 µL of each supernatant were taken to a volume of 750 µL with sodium acetate buffer (0.2 M, pH=4.75). Subsequently, 30 µL of amyloglucosidase were added and incubated at 60 °C for 45 min with constant stirring. Glucose content was measured by the Megazyme D-Glucose Assay Kit (GOPOD format; Neogen, Wicklow, Ireland).

The hydrolysis curves of samples were expressed by concentration of glucose measured in the samples with time. The hydrolysis index (HI) was calculated as the ratio between the areas under the hydrolysis curve (0–180 min) of the experimental samples and the area of reference sample (white bread). The predicted glycaemic index (pGI) was calculated using the equation proposed by Goñi *et al.* (*26*):



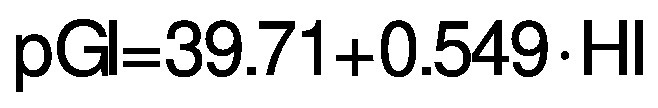



### Physical properties

#### Eccentricity, spread factor and puffiness

Ten crackers taken randomly from the batch of crackers served for the measurements of physical parameters, including mass (g), diameters of baked crackers (*d*_1_ and *d*_2_, perpendicular to each other) and thicknesses of the cracker before (*b*_1_) and after baking (*b*_2_), by Vernier calliper. The eccentricity was calculated as the ratio between *d*_1_ and *d*_2_. Spread factor was calculated as the ratio between the average diameter and average thickness (*3*). Sample puffiness (SP) was calculated as shown in the following equation:



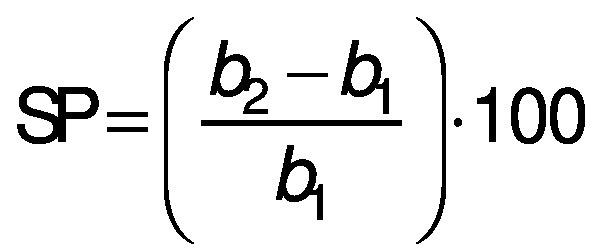



#### Textural measurements

The cracker hardness and fracturability were measured using TA-XT2 Texture Analyser (Stable Micro Systems, Godalming, UK) equipped with a 30 kg load cell and three-point bending rig (HDP/3PB). The two adjustable supports of the rig base plate were placed at 30 mm apart so as to support the sample. The upper blade was positioned to be equidistant from the two lower supports. During measurements, the upper blade descended at a speed of 1 mm/s until a 50 g contact force was detected and then travelled a distance of 5 mm through the cracker at a speed of 3.0 mm/s. Measurements were performed 24 h after baking in six replicates per batch at ambient temperature ((25±1) °C).

#### Colour measurements

The colour of the cracker top surface was measured 24 h after baking using a Chroma meter Minolta CR-400 (Konica Minolta Co., Osaka, Japan). Colour measurements were made on five randomly selected crackers per sample batch and the results were expressed as L* (lightness/darkness), a* (redness/greenness) and b* (yellowness/blueness).

The influence of the addition of different types of pumpkin seed press cake flour on the total colour difference (∆*E*) between the control and the crackers containing pumpkin flour was measured according to the following equation:



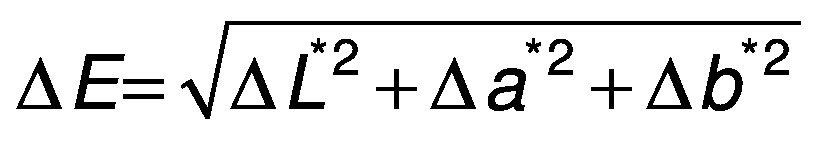



where ΔL*, Δa* and Δb* indicate the difference in L*, a* and b* values, respectively, of the crackers with virgin and cold-pressed pumpkin seed cake flour.

### Sensory evaluation

Sensory analysis of the crackers was performed by semi-trained sensory panellists (28 female and 12 male, 27–50 years of age). All panellists were recruited from the staff working at the Institute of Food Technology in Novi Sad, Serbia. Panellists evaluated liking of the cracker colour, taste, flavour, texture and overall liking on a 9-point structured hedonic scale where 1 indicates extreme disliking and 9 indicates extreme liking. Panellists worked in a sensory laboratory in individual sensory booths. They received two crackers per sample, one at a time, in closed odourless plastic containers at ambient temperature ((25±1) °C) labelled with three randomly chosen numbers and drinking water for palate cleansing. Before the analysis, all panellists received written information about the study and they signed an informed consent to participate. The study was approved by the Ethics Committee of the Institute of Food Technology in Novi Sad, University of Novi Sad, Novi Sad, Serbia (Ref. No. 175/I/13-3).

### Statistical analysis

All measurements were performed at least in triplicates if not stated differently. The mean values of replicates for analysed parameters were statistically processed using the software package XLSTAT 2019.4.2 (*27*). Analysis of variance (ANOVA) and Fisher’s least significant difference test (p<0.05) were used to determine the significance of differences between sample mean values.

## RESULTS AND DISCUSSION

### Proximate analyses of crackers

Proximate composition of crackers is presented in Table 2. Changes in the chemical composition are in the function of the gradual substitution of chickpea flour with pumpkin seed press cake flour. Although the tested samples showed significant differences in moisture content, the values were below 6%, which could be considered favourable for quality and stability of the produced crackers (*28*). The increase in the mass fraction of virgin or cold-pressed pumpkin seed cake flour increased the mass fraction of proteins, fats and ash, whereas total carbohydrate mass fraction decreased (p<0.05). The highest mass fractions of proteins and fats were observed in the crackers with 35% cold-pressed pumpkin seed cake flour. According to Regulation (EC) No. 1924/2006 (*29*), a claim that a food is a ’source of protein’ may only be made when at least 12% of the energy value of the food is provided by proteins. According to this Regulation, the control cracker and crackers with 20% of virgin or cold-pressed pumpkin seed cake flour can be labelled as protein source since 13.94% (control), 18.76% (virgin flour) and 18.91% (cold-pressed flour) of their energy value is provided by protein. In the case of crackers with higher content of virgin and cold-pressed pumpkin seed cake flour, they may bear the claim ’high protein’ as their proteins provide 22.74 and 23.98%, respectively, of the energy value. The values of total dietary fibre slightly varied among samples, and ranged from 9.17% (crackers with 20% cold-pressed pumpkin seed cake flour) to 10.22% (crackers with 35% virgin pumpkin seed cake flour). Since all cracker samples contained more than 6 g of fibre per 100 g, they can also be labelled as ’high in fibre’ (*29*).

**Table 2:** Proximate composition of different types of crackers

Parameter	Control	20VFC	35VFC	20CFC	35CFC
Proximate composition *w*(parameter)/%					
Moisture	(4.62±0.06)^d^	(5.12±0.04)^b^	(3.99±0.03)^e^	(5.61±0.06)^a^	(5.00±0.03)^c^
Carbohydrates	(42.2±0.2)^a^	(35.2±0.4)^b^	(28.8±0.0)^c^	(34.9±0.2)^b^	(26.0±0.4)^d^
Crude fat	(24.3±0.1)^d^	(24.8±0.1)^c^	(26.1±0.1)^b^	(24.9±0.1)^c^	(26.8±0.1)^a^
Protein	(16.5±0.1)^d^	(22.2±0.1)^c^	(27.3±0.0)^b^	(22.3±0.1)^c^	(28.7±0.1)^a^
Total dietary fibre	(9.95±0.13)^a^	(9.61±0.10)^b^	(10.2±0.2)^a^	(9.17±0.07)^c^	(9.42±0.17)^bc^
Ash	(2.47±0.03)^e^	(3.06±0.01)^d^	(3.67±0.03)^b^	(3.14±0.03)^c^	(3.99±0.02)^a^
*E*/kcal (kJ)	473 (1976)	472 (1971)	479 (2000)	471 (1967)	479 (1999)
*w*(mineral)/(mg/100 g)					
Zn	(3.00±0.04)^d^	(4.76±0.08)^c^	(5.90±0.06)^b^	(4.91±0.05)^c^	(6.17±0.13)^a^
Fe	(4.02±0.09)^d^	(4.67±0.11)^b^	(5.51±0.06)^ab^	(5.21±0.13)^b^	(5.79±0.24)^a^
Mg	(103±0)^e^	(170±4)^d^	(221±6)^b^	(181±3)^c^	(265±6)^a^
K	(926±17)^c^	(932±1)^bc^	(952±9)^b^	(986±11)^a^	(1007±5)^a^
Na	(810±16)^c^	(904±2)^b^	(1034±3)^a^	(827±1)^c^	(832±15)^c^
Ca	(32.4±0.5)^a^	(20.5±1.0)^b^	(17.5±0.0)^c^	(20.4±0.5)^b^	(16.0±0.2)^c^
*w*(fatty acid)/%					
Lauric acid (C12:0)	(0.18±0.02)^ab^	(0.18±0.01)^ab^	(0.17±0.02)^ab^	(0.16±0.01)^b^	(0.19±0.01)^a^
Myristic acid (C14:0)	(0.79±0.01)^a^	(0.77±0.01)^bc^	(0.75±0.01)^cd^	(0.74±0.01)^d^	(0.77±0.01)^b^
Palmitic acid (C16:0)	(36.8±0.1)^a^	(35.6±0.0)^c^	(34.9±0.0)^e^	(36.0±0.0)^b^	(35.5±0.0)^d^
Palmitoleic acid (C16:1)	(0.18±0.01)^a^	(0.17±0.02)^a^	(0.17±0.01)^a^	(0.16±0.01)^a^	(0.14±0.01)^b^
Stearic acid (C18:0)	(4.21±0.02)^c^	(4.55±0.03)^b^	(4.72±0.02)^a^	(4.56±0.01)^b^	(4.74±0.02)^a^
Oleic acid (C18:1n9c)	(38.3±0.1)^c^	(38.6±0.1)^ab^	(38.7±0.1)^a^	(38.5±0.1)^abc^	(38.4±0.1)^bc^
Linoleic acid (C18:2n6c)	(18.0±0.1)^d^	(18.6±0.1)^b^	(18.9±0.1)^a^	(18.5±0.0)^c^	(18.9±0.1)^a^
Arachidic acid (C20:0)	(0.44±0.01)^b^	(0.44±0.01)^b^	(0.52±0.02)^a^	(0.46±0.01)^b^	(0.46±0.01)^b^
α-Linolenic acid (C18:3n3)	(0.77±0.03)^a^	(0.74±0.01)^a^	(0.74±0.01)^a^	(0.68±0.02)^b^	(0.67±0.01)^b^
Eicosanoic acid (C20:1n9)	(0.26±0.01)^bc^	(0.33±0.01)^a^	(0.26±0.01)^b^	(0.24±0.02)^c^	(0.25±0.00)^bc^
Total SFA	42.5	41.6	41.1	41.9	41.7
Total MUFA	38.8	39.1	39.2	38.9	38.8
Total PUFA	18.8	19.4	19.7	19.1	19.5
PUFA/SFA	0.44	0.47	0.48	0.46	0.47

Control=chickpea crackers, 20VFC and 35VFC=crackers with 20 or 35% of chickpea flour replaced with pumpkin seed press cake flour obtained after virgin pumpkin oil extraction, 20CFC and 35CFC=crackers with 20 or 35% chickpea flour replaced with pumpkin seed press cake flour obtained after cold-pressed oil extraction, SFA=saturated fatty acids, MUFA=monounsaturated fatty acids, PUFA=polyunsaturated fatty acids. Values are presented as mean±standard deviation (*N*=3). Mean values in the same row with different letters in superscript are statistically different (p≤0.05) according to Fisher’s LSD test

### Mineral content of crackers

Potassium was the most abundant macroelement in all cracker samples, followed by calcium and magnesium (Table 2). In comparison with gluten-free crackers enriched with hemp flour and decaffeinated green tea leaves (*30*), crackers produced in this study had higher mass fraction of all tested minerals except calcium. Missbach *et al.* (*31*) found that potassium mass fraction was significantly lower in gluten-free snacks than in their gluten counterparts ((190.4±160.0) mg/100 g compared to (247.5±130.0) mg/100 g), while in this study potassium mass fraction was in the range from 926 to 1007 mg/100 g. Substitution of chickpea flour with virgin and cold-pressed pumpkin seed cake flour significantly (p<0.05) altered the mass fraction of minerals. With an increase in the amount of pumpkin seed cake flour, the mass fraction of almost all minerals progressively rose. The exception is calcium, with the highest mass fraction recorded in the control sample. To declare a product as a product rich in certain minerals, it must contain 15% of dietary reference values in 100 g of the product (*32*). Based on the report of the European Food Safety Authority (*32*), all samples are a significant source of Zn, Fe, Mg and K. Bearing in mind that gluten-free products have low amount of minerals, which can be harmful for patients suffering from coeliac disease (*33*), the selection of these raw materials for cracker production is justified.

### Fatty acid composition of crackers

The analysis of fatty acid profile has shown that the dominant fatty acids in all the samples were palmitic (C16:0), oleic (C18:1n9c) and linoleic (C18:2n6c) acids (Table 2). The mass fraction of linoleic acid increased as a consequence of increasing the amount of pumpkin seed cake flour. In the case of α-linolenic acid, the presence of cold-pressed pumpkin seed cake flour in the cracker formulation affected its reduction. In a balanced diet, the recommended ratio of PUFA/SFA should be above 0.4 (*34*). In all samples the ratio was over 0.4, which indicates that the choice of ingredients contributes to favourable fatty acid composition. Considering the MUFA intake, the Adult Treatment Panel III (*35*) recommended up to 20% of energy intake as MUFA. Regarding the results obtained for fatty acid composition, the content of MUFA is in accordance with the proposed recommendation.

### Total phenolic content and antioxidant activity

Polyphenols, secondary plant metabolites, possess different biological activities such as ability to inhibit reactive oxygen and nitrogen species, oxidative enzymes, to activate antioxidant enzymes and act as free radical scavengers (*36*). Values of total phenolic content (TPC), expressed as GAE, were in the range from 0.72 in the control sample to 1.06 mg/g in the sample with 35% cold-pressed pumpkin seed cake flour (Table 3). The pumpkin oil cake incorporated crackers showed significant (p<0.05) increase in the TPC.

**Table 3:** Total phenolic content (expressed as gallic acid mass fraction), antioxidant activity (in mM Trolox per g of sample), hydrolysis index and predicted glycaemic index of crackers

Parameter	Control	20VFC	35VFC	20CFC	35CFC
*b*(Trolox)/(mM/g)	(8.0±0.4)^d^	(12.0±0.2)^b^	(11.9±0.2)^b^	(11.5±0.3)^b^	(17.0±0.4)^a^
*w*(galic acid)/(mg/g)	(0.72±0.00)^d^	(0.84±0.00)^bc^	(0.82±0.03)^c^	(0.88±0.01)^b^	(1.06±0.00)^a^
Hydrolysis index	(51.2±0.1)^a^	(38.8±0.3)^d^	(36.8±0.1)^e^	(40.1±0.2)^b^	(39.5±0.4)^c^
Predicted glycaemic index	(67.8±0.1)^a^	(61.0±0.6)^b^	(59.9±0.1)^c^	(61.7±0.5)^b^	(61.4±0.3)^b^

Control=chickpea crackers, 20VFC and 35VFC=crackers with 20 or 35% of chickpea flour replaced with pumpkin seed press cake flour obtained after virgin pumpkin oil extraction, 20CFC and 35CFC=crackers with 20 or 35% chickpea flour replaced with pumpkin seed press cake flour obtained after cold-pressed oil extraction. Values are presented as mean±standard deviation (*N*=3). Mean values in the same row with different letters in superscript are statistically different (p≤0.05) according to Fisher’s LSD test

The antioxidant activity of the formulated crackers determined by ABTS assay revealed a similar trend to that found in the Folin-Ciocalteu assay (Table 3). The addition of pumpkin seed press cake flour resulted in higher values of antioxidant activity, expressed in TE, ranging from 11.5 to 17.0 mM/g. The higher values of antioxidant activity in the 35CFC might result from the initial presence of phenolic compounds in the cold-pressed cake, while in the virgin pumpkin seed cake flour the amount of phenolic compounds, which are known to be thermolabile components, can be reduced due to the thermal treatment of the seeds before oil extraction. In this respect, the addition of cold-pressed pumpkin seed cake flour to cracker formulation enhanced their antioxidant potential, which could be beneficial for the stability against oxidative damage during storage.

### Glycaemic index of crackers

**Fig. 1:**
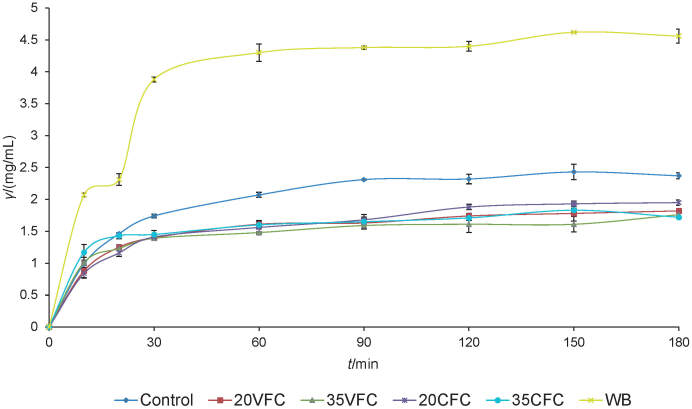
Glucose concentration as a function of time during the second intestinal phase, relevant to the examined crackers. Control=chickpea crackers, 20VFC and 35VFC=crackers with 20 or 35% of chickpea flour replaced with pumpkin seed press cake flour obtained after virgin pumpkin oil extraction, 20CFC and 35CFC=crackers with 20 or 35% chickpea flour replaced with pumpkin seed press cake flour remaining after cold-pressed oil extraction, WB=commercial white bread

Compared to their gluten-containing counterparts, gluten-free baked products are often considered as products with high glycaemic index. This is due to the prevalence of carbohydrate components of commonly used gluten-free flour such as rice and maize flour. Moreover, there is a theoretical presumption that in the absence of gluten protein network that usually surrounds the starch granules, they become more susceptible to the action of amylolytic enzymes, which additionally contribute to the higher glycaemic index (*37*, *38*). Considering the complex matrix characteristics of the bakery products, in addition to the above, glycaemic index is highly influenced by several factors such as the type of starch (the amylose/amylopectin ratio), physical entrapment of starch molecules within food, food formulations and processing, and by the presence of other ingredients, such as sugars, fat, protein, dietary fibre and anti-nutrients (*5*, *37*, *39*, *40*). Accordingly, it is of great importance to introduce raw materials with the potential of reducing glycaemic index in the manufacturing of gluten-free products. The starch hydrolysis curves during the second intestinal phase of *in vitro* digestion of all produced crackers along with white bread are presented in Fig. 1. The hydrolysis index, calculated from the starch hydrolysis curves, and the corresponding predicted glycaemic index (pGI) are shown in Table 3. Glucose concentration increased during *in vitro* digestion in all samples, with a sharp increase during the first 30 min. As expected, the maximum glucose concentration was recorded in white bread, which served as a benchmark, while all the crackers had significantly lower glucose concentration with maximum occurring at 60 min and remaining almost the same during all the examined digestion time. According to the available literature data, the predicted glycaemic index for gluten-free cookies and crackers with the addition of pulse flour ranged from low (<55) to intermediate (55–70) (*5*, *41*). The control cracker had a glycaemic index of 67.8±0.3, while substitution of chickpea flour by pumpkin seed press cake flour at 20 or 35% significantly (p<0.05) reduced cracker glycaemic index to approx. 60. The results obtained in this study showed that with the selected combination of raw materials, it is feasible to obtain gluten-free crackers with a moderate glycaemic index (*42*), where the use of flour from pumpkin seed cakes allows a further reduction in the predicted glycaemic index.

### Physical properties of crackers

#### Eccentricity, spread factor and puffiness of crackers

Physical properties of crackers are summarized in Table 4. There were no significant differences between the eccentricity and spread factor of samples. However, an increase in the spread factor values is observed with the substitution of chickpea flour with virgin or cold-pressed pumpkin seed cake flour. Compared to the control sample, crackers with the two types of pumpkin seed cake flour have higher mass values. Furthermore, addition of pumpkin seed cake flour affected the puffiness of samples. Significant dimensional changes are typical in the formulation of cookies with wheat flour and composite blends as well as in gluten-free formulations for cookies based on rice or maize flour as a consequence of incorporation of high protein raw materials (*15*, *43*). Since all the tested samples had a high protein and fibre content, it can be assumed that the used raw materials caused a higher amount of water retention in the dough system, which limited the free water and thus did not affect significant changes in dimensional parameters. This is in accordance with the research of Mancebo *et al.* (*44*), where the presence of flour with higher protein content in gluten-free formulations had no significant influence on the spread ratio of cookies. Although a certain increase in cookie diameter and spread ratio is desirable for better quality (*43*), the advantage of the uniform cookie dimensions could be reflected in the simplified control of the product packaging.

**Table 4:** Physical properties of crackers

Parameter	Control	20VFC		35VFC		20CFC	35CFC
*m*/g	(4.85±0.05)^b^	(4.89±0.06)^ab^		(4.98±0.08)^a^		(5.0±0.1)^a^	(4.95±0.08)^ab^
Eccentricity/%	(1.00±0.01)^a^	(0.99±0.00)^a^		(0.99±0.01)^a^		(1.00±0.01)^a^	(0.99±0.00)^a^
Spread factor/%	(9.9±0.2)^a^	(10.1±0.2)^a^		(10.1±0.1)^a^		(9.9±0.1)^a^	(9.98±0.06)^a^
Puffiness/%	(28.9±3.4)^a^	(25.2±4.5)^ab^		(23.4±3.4)^ab^		(21.6±8.0)^b^	(23.8±3.8)^ab^
Colour properties							
L*	(69.0±0.3)^a^		(50.5±0.7)^d^	(46.2±0.5)^e^	(57.1±0.7)^b^		(51.4±0.9)^c^
a*	(5.2±0.2)^a^		(4.2±0.2)^b^	(4.4±0.2)^b^	(1.9±0.2)^c^		(1.2±0.2)^c^
b*	(34.6±0.3)^a^		(19.1±0.7)^c^	(15.1±0.6)^d^	(23.9±1.0)^b^		(18.7±0.8)^c^
Δ*E*			(24.2±1.0)^b^	(30.0±0.8)^a^	(16.4±1.2)^c^		(23.9±1.2)^b^
Textural properties							
Hardness/kg	(2.8±0.7)^ab^		(3.3±0.5)^ab^	(3.5±0.4)^a^	(2.6±0.5)^b^		(3.0±0.5)^ab^
Fracturability/mm	(2.2±0.3)^a^		(1.0±0.1)^b^	(0.95±0.09)^b^	(2.2±0.2)^a^		(2.0±0.2)^a^

Control=chickpea crackers, 20VFC and 35VFC=crackers with 20 or 35% of chickpea flour replaced with pumpkin seed press cake flour obtained after virgin pumpkin oil extraction, 20CFC and 35CFC=crackers with 20 or 35% chickpea flour replaced with pumpkin seed press cake flour remaining after cold-pressed oil extraction, L*=lightness/darkness, a*=redness/greenness, b*=yellowness/blueness, Δ*E*=total colour difference. Values are presented as mean±standard deviation (*N*=3). Mean values in the same row with different superscript letters are statistically different (p≤0.05) according to Fisher’s LSD test

#### Colour of crackers

**Fig. S1:**
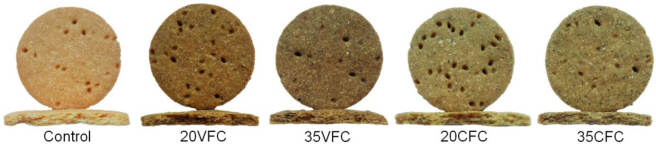
Appearance of the gluten-free crackers. Control=chickpea crackers, 20VFC and 35VFC=crackers with 20 or 35% of chickpea flour replaced with pumpkin seed press cake flour obtained after virgin pumpkin oil extraction, 20CFC and 35CFC=crackers with 20 or 35% chickpea flour replaced with pumpkin seed press cake flour remaining after cold-pressed oil extraction

Crackers containing pumpkin seed cake flour, regardless of type, had unusual colour for this type of product, as can be observed in Fig. S1. Reduction of the lightness parameter L* is observable with increasing the mass fractions of both types of pumpkin seed cake flour (Table 4). Parameters a* and b* were also reduced, indicating an increase in green colour and decrease in the yellowness of biscuits. The green colour of pumpkin seed cake flour derives from protochlorophyll found in chlorenchyma, the dark green layer around cotyledons of pumpkin (*45*). The results showed that crackers containing virgin pumpkin seed press cake flour were more red and less yellow than the crackers containing cold-pressed pumpkin seed flour. This could be related to the higher pigment degradation with the thermal treatment of the seeds before oil extraction and then baking of the cracker. The total colour difference (Δ*E*) was highly influenced by the amount of pumpkin seed press cake flour.

#### Hardness and fracturability of crackers

Textural properties of crackers expressed as hardness (maximum peak force recorded from force/distance curve) and fracturability (peak distance which represents the distance the cracker will deform before breaking) are listed in Table 4. According to the obtained results, partial replacement of chickpea flour with cold-pressed pumpkin seed cake flour did not influence cracker texture although there was significant difference in the protein and starch mass fractions of the crackers (Table 2). Similar hardness and fracturability of control and crackers with cold-pressed pumpkin seed cake flour were probably influenced by the combined effect of gelatinized starch granules embedded in the protein matrix, where the former were more dominant in the control sample, while the latter contributed to the texture formation in the samples with cold-pressed pumpkin seed cake flour. On the contrary, incorporation of virgin pumpkin seed cake flour led to an increase in cracker hardness and decrease in its fracturability. Unlike cold-pressed seed cake flour, the virgin pumpkin seed cake flour was thermally treated during oil extraction, which affected protein denaturation to a higher degree and consequently led to higher water retention in the cracker dough (*46*). Higher content of retained water in these formulations than in those with cold-pressed pumpkin seed cake flour produced stiffer dough and subsequently harder crackers (*47*). However, no significant difference in cracker hardness and fracturability was observed with the increase of mass fractions of both types of pumpkin seed flour.

### Sensory analysis (overall liking, colour, taste, texture and flavour) of crackers

**Fig. 2:**
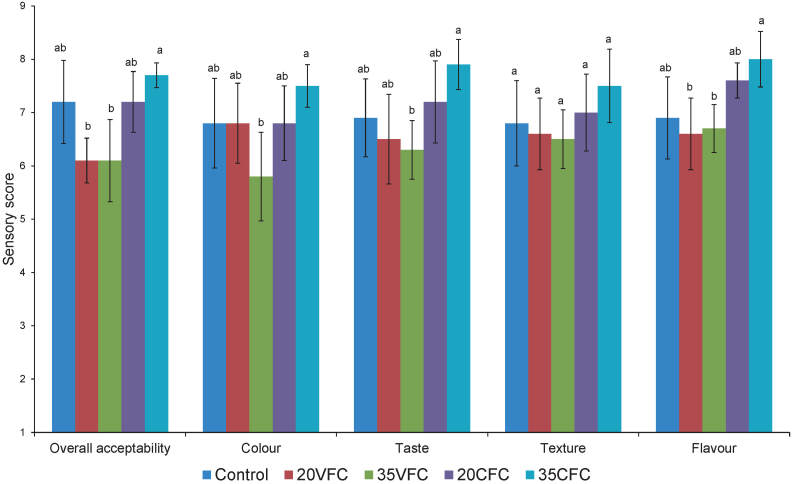
Sensory evaluation scores for overall liking, liking of colour, taste, texture and flavour of gluten-free crackers. Control=chickpea crackers, 20VFC and 35VFC=crackers with 20 or 35% of chickpea flour replaced with pumpkin seed press cake flour obtained after virgin pumpkin oil extraction, 20CFC and 35CFC=crackers with 20 or 35% chickpea flour replaced with pumpkin seed press cake flour remaining after cold-pressed oil extraction

Results of sensory study showed that all sensory scores for crackers were in the range from like slightly to like very much (Fig. 2). Replacement of chickpea flour with virgin pumpkin seed cake flour slightly decreased, while replacement with cold-pressed pumpkin seed cake flour significantly increased overall liking of crackers, especially their taste and flavour. Cracker 35CFC was superior in terms of all analysed sensory properties, followed by the 20CFC. Cracker 35VFC was the least acceptable. In their study, Kaur and Sharma (*48*) showed that cookies supplemented with raw pumpkin seed flour were more acceptable than the cookies supplemented with roasted pumpkin seed flour, which is in agreement with the results presented in this study. Although colour differences between samples were clearly visible, the colour liking among samples was not significant (p<0.05), except between samples 35VFC and 35CFC (p>0.05). Liking scores were in the range from ’slight liking’ (5.80) for 35VFC to ’like very much’ (7.50) for 35CFC. Han *et al.* (*1*) studied the effect of various types of pulse flour and different mass fractions (desi chickpea, green lentil, red lentil, pinto bean, navy bean and yellow pea) on the development of gluten-free pulse-based cracker snacks, and similar to our findings, they found no statistical difference in colour acceptability scores that were in the range from 6.0 (for crackers containing navy bean flour) to 6.7 (for crackers containing pea protein isolate). Moreover, they concluded that within the evaluated range of samples, the colour was not barrier to product acceptability.

## CONCLUSIONS

The present study revealed that the combination of chickpea and pumpkin seed press cake flour could be successfully utilised in the production of gluten-free crackers without the use of conventional gluten-free starch-rich ingredients. These selected raw materials had multiple benefits which are reflected in the increased nutritional quality of final product, including increased protein and dietary fibre content, improved mineral and fatty acid profile, enhanced total phenolic content and antioxidant activity compared to the control sample. The selected combination of raw materials used in this study allows the production of gluten-free crackers with a moderate glycaemic index. Moreover, it was shown that cold-pressed pumpkin seed cake flour would perform better than virgin pumpkin seed cake flour in terms of product texture and overall acceptability. Further research should be focused on the cracker shelf life assessment but also on the safety aspects of this novel food product.
